# A Spatio-Temporal Motion Network for Action Recognition Based on Spatial Attention

**DOI:** 10.3390/e24030368

**Published:** 2022-03-04

**Authors:** Qi Yang, Tongwei Lu, Huabing Zhou

**Affiliations:** 1 School of Computer Science and Engineering, Wuhan Institute of Technology, Wuhan 430205, China; crush_yq@yeah.net (Q.Y.); zhou@wit.edu.cn (H.Z.); 2 Hubei Key Laboratory of Intelligent Robot, Wuhan Institute of Technology, Wuhan 430205, China

**Keywords:** temporal modeling, spatio-temporal motion, group convolution, spatial attention

## Abstract

Temporal modeling is the key for action recognition in videos, but traditional 2D CNNs do not capture temporal relationships well. 3D CNNs can achieve good performance, but are computationally intensive and not well practiced on existing devices. Based on these problems, we design a generic and effective module called spatio-temporal motion network (SMNet). SMNet maintains the complexity of 2D and reduces the computational effort of the algorithm while achieving performance comparable to 3D CNNs. SMNet contains a spatio-temporal excitation module (SE) and a motion excitation module (ME). The SE module uses group convolution to fuse temporal information to reduce the number of parameters in the network, and uses spatial attention to extract spatial information. The ME module uses the difference between adjacent frames to extract feature-level motion patterns between adjacent frames, which can effectively encode motion features and help identify actions efficiently. We use ResNet-50 as the backbone network and insert SMNet into the residual blocks to form a simple and effective action network. The experiment results on three datasets, namely Something-Something V1, Something-Something V2, and Kinetics-400, show that it out performs state-of-the-arts motion recognition networks.

## 1. Introduction

Action recognition is most important for video understanding, which aims to enable the computer to accurately understand the video content and classify the video. The video action recognition method maps the motion information and spatial information of the original video data to the feature space to obtain the feature expression of the video, and realizes the accurate classification of the action in the video according to the feature descriptor. Therefore, how to extract the action information that can accurately represent the video content is the key problem in the task of video action recognition. Existing action recognition is divided into 2D-based action recognition and 3D-based action recognition. However, 2D-based action recognition mostly extracts motion features through optical flow, which requires additional cost. 3D-based action recognition has higher performance than 2D, but is computationally intensive and not well suited for practical applications. Therefore, there is a need for an operational action recognition method in a practical environment.

The existing action recognition methods mainly focus on the extraction of motion features and spatio-temporal features. For the extraction of spatio-temporal information, the existing methods [1,2,3] directly use 2D CNN for feature extraction of RGB images, but these methods lack information on the temporal sequence. Another approach is to use a 3D CNN [4] to extract features such that each feature map in the convolutional layer is connected to multiple adjacent consecutive frames in the previous layer [5]. Such an approach can capture spatio-temporal features well, and allow temporal information to be preserved, but extending the convolution kernel from 2D to 3D will inevitably increase by an order of magnitude, making the computational effort expand exponentially, which limits its real time application. To solve this problem, we use a spatial attention mechanism to focus on the part that people are interested in, thus giving more weight to this part in the training. By introducing weighting information, we can better focus on the more important features and suppress unnecessary features. In addition, using group convolution for temporal information extraction in the temporal dimension allows for a relative reduction in computational effort.

For the extraction of motion information, the existing methods [6,7] extract optical flow [8,9] information manually and then feed the optical flow information into a 2D CNN-based two-stream network for feature extraction, but the optical flow lacks the ability to capture long-term temporal relationships. In addition, optical flow needs additional extraction, which is very expensive in time complexity and space complexity, limiting its real time application. Moreover, in the two-stream network, the feature learning of spatio-temporal and motion information is isolated, and only the last stage of the network processes the information fusion. To solve these problems, we use the ME module for motion information extraction.The ME module uses RGB differences in successive frames to extract feature-level motion patterns and thus build a motion feature model. Our goal is simply the search for a motion representation, not precise motion information between consecutive frames. Therefore, we will only use RGB frames and not involve any pre-computed optical flow.

**Figure 1:**
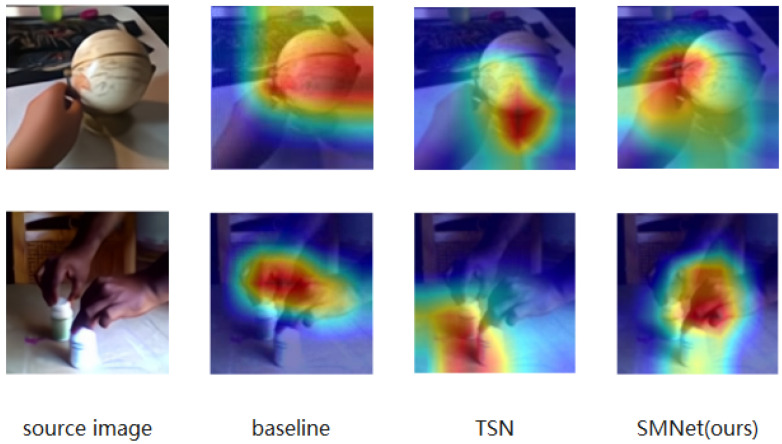
We used Grad-CAM to visualize our image features on baseline, TSN and SMNet (ours).

Inspired by the above observations, we designed a simple and effective SMNet module to integrate spatio-temporal and motion features into a unified 2D CNN framework without any three-dimensional convolution and optical flow precomputation. Given an input feature graph, we use the ME module to calculate the feature level motion representation between adjacent frames. Inspired by CBAM [10], our SE module extracts features based on spatial dimension to represent spatial information and characterize the channel information of video frames by means of group convolution. We fuse the two modules by connecting them side-by-side and combine them into SMNet. SMNet blocks can be easily inserted into the existing ResNet [11] architecture to replace the original residual blocks without adding too many parameters. As shown in Figure 1, we visualized baseline [1], TSN [2] and our method for feature extraction of some actions. We can observe that baseline cannot well recognize the region where the action is located, while TSN only focuses on recognizing objects independently rather than reasoning about an action. Compared with baseline and TSN, our proposed spatio-temporal motion focuses on people’s actions on the basis of accurately recognizing the motion region, which can be seen in the last column. In short, our contribution has three aspects:

(1) We propose a spatio-temporal information module based on attention mechanism to model temporal and spatial information, and use the form of group convolution to fuse temporal information, which improves the recognition ability of the network without adding too much computation.
(2) We propose a spatio-temporal motion network, which combines spatiotemporal information and motion information, which can be easily integrated into ResNet network, and can recognize action simply and efficiently.
(3) Our SMNet performs experiments on different datasets such as Something-Something V1, Something-Something V2 and Kinetics-400, and obtains some good results.

## 2. Ralated Works

In this section, we give a brief introduction of 3D CNNs, temporal dependency in action recognition, attention mechanism and bilinear pooling, respectively.

### 2.1. 2D Network

Action recognition is a fundamental problem in the task of video, and has been widely used in recent years in video retrieval, surveillance, human-computer interaction, virtual reality, smart homes and intelligent security, and has been subject to intensive academic research. In recent years, with the development of deep learning, there have been many action recognition methods, the most significant of which are the extraction of spatio-temporal features and motion features. The two-stream network proposed by Simonyan et al. [1,4] used a two-branch network architecture to capture the spatial and temporal information of the video respectively. The spatial domain uses RGB images as input to extract appearance features and the time domain uses optical flow information as input to extract temporal features. Feichtenhofer et al. [6,11] followed the architecture of two-stream network and investigated the fusion strategy of two-stream. The authors improved the fusion strategy in the spatial domain and time domain, proposed five different fusion schemes in the problem of fusion of spatial domain networks and time domain networks, and introduced three methods in the problem of time domain fusion. TSN [2] proposed a specific network for video action recognition based on a long-range temporal structure. First, TSN uses a sparse temporal sampling strategy and a video supervision-based strategy to segment the video in the time domain and then randomly select segments as network input. Then, training is performed using cross-practice, regularisation techniques and data expansion, and finally, fusion is performed by weighted averaging in the latter part of the network. However, all these methods require additional computational and storage costs to process the optical stream. TSM [12] proposed shifting part of the channel along the time dimension to facilitate information exchange between adjacent frames. It is the first to introduce temporal modelling into a 2D CNN-based framework and to embed the partial channel shifting operation into 2D CNNs. However, TSM [13] lacks an explicit model of action time modelling, such as the difference between adjacent frames. All these networks require additional cost and computational effort to obtain and store optical flow information, which is cost prohibitive. In contrast, our approach does not require additional optical flow information as input, only RGB images as input to the network, which greatly reduces the additional cost and is very beneficial for practical applications.

Recently, some work has proposed that some modules that can model motion and temporal information can be embedded in 2D CNN. Examples include MFNet [14], TEINet [15] and TEA [16], which have been shown to be effective on the ResNet architecture. STM [17] models motion features at the feature level, proposing a block that models both spatio-temporal and motion information instead of a normal residual block. Specifically, given a feature map, spatio-temporal and motion features are obtained separately, and the two features are fused to obtain an STM [17] block. the STM [17] block can be easily inserted into ResNet to replace the original residual block. However, it still cannot strike a good balance between accuracy and computational effort, and the extraction of temporal information is unsatisfactory. Our network continues this design, replacing the original residual blocks of ResNet with our SMNet blocks for the design, and using group convolution to reduce the number of partial parameters. At the end of the network, the fusion strategy is used to integrate the feature information.

### 2.2. 3D Network

Another type of action recognition is modelled using 3D CNNs. C3D [5] simply used 3D convolutional kernels to process video, and identified 3 × 3 × 3 as the optimal convolutional kernel size through comparative experiments. The I3D proposed by Carreira et al. [18] combined a 3D convolutional network with a two-stream network. The convolutional network applies the Inception-V1 model, extending the convolutional kernels in the original network to 3D convolutional kernels, and directly using the parameters of 2D convolutional kernels already pre-trained on ImageNet to initialize the parameters and pre-train them on the Kinetics database to improve the performance of the network. Based on this, Diba et al. [19] proposed T3D to transfer parameters from a pre-trained 2D convolutional network to a randomly initialised 3D network for stable weight initialisation. T3D replaced the convolutional kernel in DenseNet [20] with a 3D convolutional kernel, and proposed a TTL layer (Temporal Transition Layer) which is able to capture information at different times using convolution at different scales, and finally embeds the TTL layer into DenseNet. The P3D residual network proposed by Qiu et al. [21] reduces the parameters of the network. P3D used the network framework of ResNet, using 1 × 3 × 3 convolution (to obtain spatial dimensional features) and 3 × 1 × 1 convolution (to obtain temporal dimensional features) in cascade or parallel instead of the original 3D convolution of 3 × 3 × 3, and a deeper network is designed to apply all three P3D structures into it. Slowfast [22] proposed a combined fast-slow network for video classification, with a model consisting of two parts: a Slow pathway running at low frame rates to capture spatial semantic information, and a Fast pathway running at high frame rates to capture action information with good temporal resolution. The X3D [23] is a family of efficient video networks that progressively expand a tiny 2D image classification architecture along multiple network axes, in space, time, width and depth. Inspired by feature selection methods in machine learning, a simple stepwise network expansion approach is employed that expands a single axis in each step, such that good accuracy to complexity trade-off is achieved. While these networks extract the temporal information of the video well, extending the convolutional kernel from 2D to 3D would inevitably multiply the computational effort of the network, which is not conducive to practical applications of the network. Our network uses temporal group convolution to extract temporal information, which makes it as accurate as some 3D networks, but much less computationally intensive.

### 2.3. Attention Mechanism

Attention mechanism is a data processing method in machine learning, which is widely used in various types of machine learning tasks. It optimizes the original features by extracting the attention scores of each part of the feature expression and taking them as the fusion weight. The feature expression optimized by attention mechanism can effectively highlight the important components in the original data, so as to make it more suitable for the requirements of corresponding tasks. Jaderberg et al. [24] propose an attention mechanism that allows the spatial information in the original image to be transformed into another space and retains the key information. Specifically, they propose a module called the spatial transformer, which transforms the spatial domain information in the image into the corresponding spatial transformation, so that the key information can be extracted. Jie et al. [25] They wish to explicitly model the interdependencies between feature channels. A new "feature rescaling" strategy is used. Specifically, a learning process is used to automatically obtain the importance of each feature channel, and then to promote useful features and suppress features that are less useful for the task at hand according to this importance. Wang et al. [26] propose “Residual Attention Network”, a convolutional neural network using attention mechanism which can incorporate with state-of-art feed forward network architecture in an end-to-end training fashion. Woo S et al. [10] propose a simple yet effective attention module for feed-forward convolutional neural networks. Given an intermediate feature map, the module sequentially infers attention maps along two separate dimensions, channel and spatial, then the attention maps are multiplied to the input feature map for adaptive feature refinement. Based on this, our network incorporates a spatial attention mechanism to focus more attention on human actions rather than background.

### 2.4. Group Convolution

Vanilla Convolution essentially consists of a set of convolution kernels with parameters, which are used to extract local features from an image. The process of convolution actually involves traversing the filter from top to bottom and left to right, weighting the elements at the corresponding positions each time they are matched and outputting them. For feature extraction, the traditional convolution is the process of computing the overall convolution of the input image, sliding over the image using the convolution kernel, multiplying the pixel grey values on the image points with the values on the corresponding convolution kernel, then adding all the multiplied values as the grey values of the pixels on the image corresponding to the middle pixel of the convolution kernel, and finally sliding over all the images. However, such convolution is easy to increase the amount of calculation and parameters on large networks, which makes the iterative learning process more cumbersome. Krizhevsky et al. [27] first introduced the concept of grouped convolution and used it in practice, resulting in a significant reduction in model parameters and video memory occupation. Based on this, our model uses group convolution on temporal sequences to reduce the model parameters and extract temporal information.

## 3. Approach

In this section, we describe our spatio-temporal motion module (SMNet) in detail. First, we describe our overall network framework. Then, we perform a detailed analysis and modification of our network. For more details about our network, please refer to Section 3.1 and Section 3.2.

### 3.1. SMNet Network

**Figure 2:**
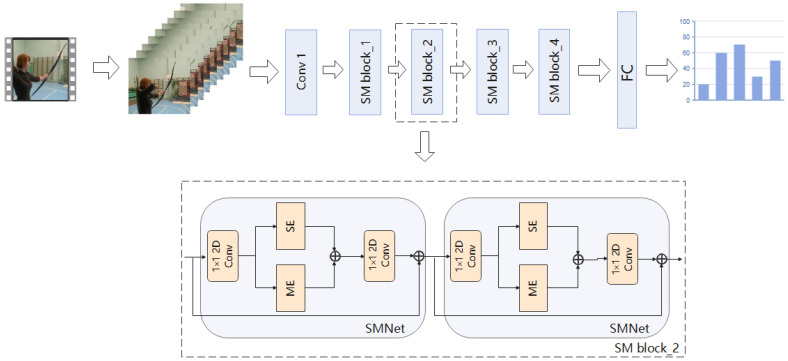
In the overall model framework. We use the sparse time sampling strategy proposed by TSN to sample the input video. Given a video, we divide it into N segments, and then randomly select a frame from each segment to form an input sequence. In order to model spatio-temporal movements, we have designed the SMNet module. SMNet contains a SE module, which is designed to extract accurate spatio-temporal information, and a ME module, which is designed to extract fine motion information. In order to improve the accuracy of the model, we superimposed several SMNet modules (four SMNet modules were superimposed in the experiment) in the residual block of ResNet-50. Details are given in Section 4.4 Ablation experiments.

We design a 2D action recognition network, which only takes RGB image as input, extracts different information from the image for action recognition, and avoids the influence of optical flow. Because the importance of different spatial location information in video frames is different, we introduce spatial attention mechanism into our video action recognition task. In the existing ordinary convolution, a convolution kernel can only extract one feature graph, so we introduce group convolution into our network, which can generate multiple feature graphs with the same amount of computation and parameters to obtain more feature information. Our model framework is shown in Figure 2. We use the sparse time sampling strategy proposed by TSN [2] to sample the input video. First, given a video, we divide it into N segments, and then randomly select a frame from each segment to form an input sequence. For the modelling of spatio-temporal motion, we build it based on ResNet-50 [11] and overlay multiple SMNet modules in the residual block of ResNet-50. The SMNet block contains a spatio-temporal excitation module (SE) to extract spatio-temporal information, and a motion excitation module (ME) to extract motion information.

### 3.2. Network Analysis

#### 3.2.1. Sampling Strategy

We use a sparse sampling strategy for input frame selection, a part of which we can see proposed in the [2] paper. Since our actions are continuous, after cropping the video into frames, we can find that the frames in some adjacent frames are almost similar. So, we segment the video frames in the chronological order in which the action takes place. Once the frames have been segmented, we can see that each segment contains approximately the same number of frames. So, in order to get a better appearance and to reduce the redundancy of the input video frames, we randomly select frames within each segment to form our input sequence. In this case, each frame in our input sequence is more or less distinct, and then the input is trained. The details of this are discussed in detail in the [1] paper. Therefore, it is suitable to use a sparse sampling strategy to randomly select input frames for the construction of the input sequence.

#### 3.2.2. SE

**Figure 3:**
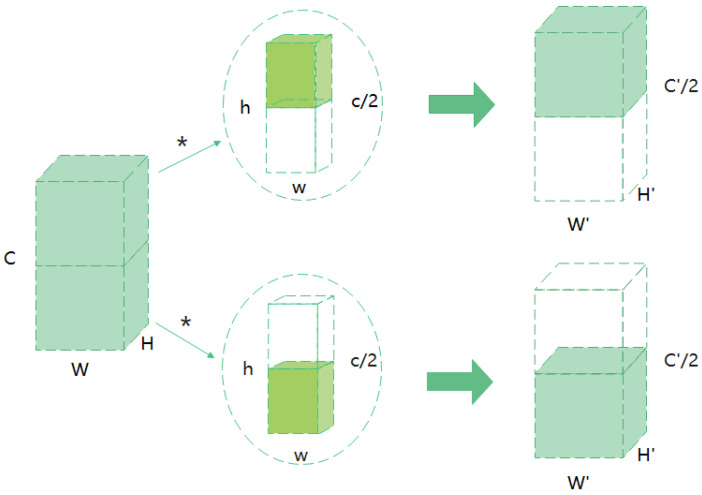
Structure of group convolution. We group the input feature maps (W × H × C ), and each convolution kernel is divided into groups accordingly. Here both the feature map and the convolution kernel are divided into two groups. Then, we perform convolution calculation to obtain the output data of each group, and then concatenate them. * represents a convolution operation.

The SE module extracts information on the time domain by group convolution. we propose to use group convolution [27], as shown in Figure 3. Compared with ordinary convolution, group convolution has some inherent advantages. Firstly, group convolution pays more attention to learning independent convolution kernel on different channels. We all know that semantic information is different on different channels, Therefore, such operation can make the information on each channel independent, which is convenient for better timing information extraction. Secondly, group convolution can greatly reduce the parameters of the model, and can generate multiple feature maps when using the same number of parameters and computation, so that our network can encode more information. We group the input feature map, and each convolution kernel is also divided into groups accordingly. The convolution kernel of each group is used to convolute with the input data part in the corresponding group to obtain the output data of each group, and then combine them through concatenate to form a feature map, so as to reduce the amount of network parameters or increase the feature map and improve the feature representation.

**Figure 4:**
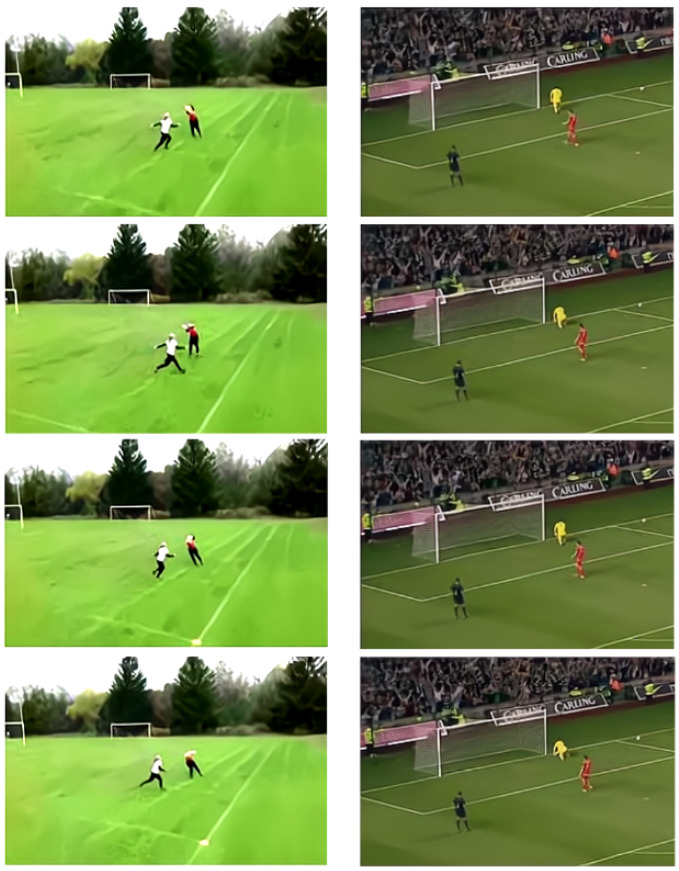
Different actions with similar backgrounds.Frisbee Catch and Soccer Penalty.

**Figure 5:**
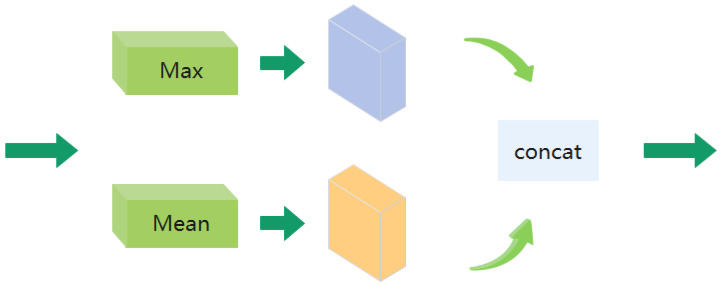
Spatial attention structure module. We perform average pooling and maximum pooling along the channel direction, and concatenate the two feature maps.

The SE module introduces a spatial attention mechanism for information extraction on the spatial domain. Because in the video frame, we often need to pay attention to the action of the human body and ignore the influence of the chaotic background. Furthermore, we take into account the varying importance of spatial location information in images, so we introduce a spatial attention module to train to obtain a spatial weight matrix. The attention mechanism is introduced to increase expressiveness by focusing on the more important features and suppressing unnecessary ones, which has a significant effect in distinguishing between different actions with similar backgrounds as shown in Figure 4. Therefore, we use the attention mechanism in the spatial domain to extract spatial information. As shown in Figure 5, inspired by [28], we believe that applying pooling operation along the channel axis can effectively highlight the areas containing key information. So, we perform average pooling and maximum pooling along the channel direction to obtain two two-dimensional feature maps. The obtained two feature maps are concatenated in the dimensional direction to obtain the spliced feature map. The channel of the new feature map is the sum of the original two feature map channels. For the spliced feature map, the spatial attention map is generated by convolution layer, and then multiplied with the original features to obtain new features.

**Figure 6:**
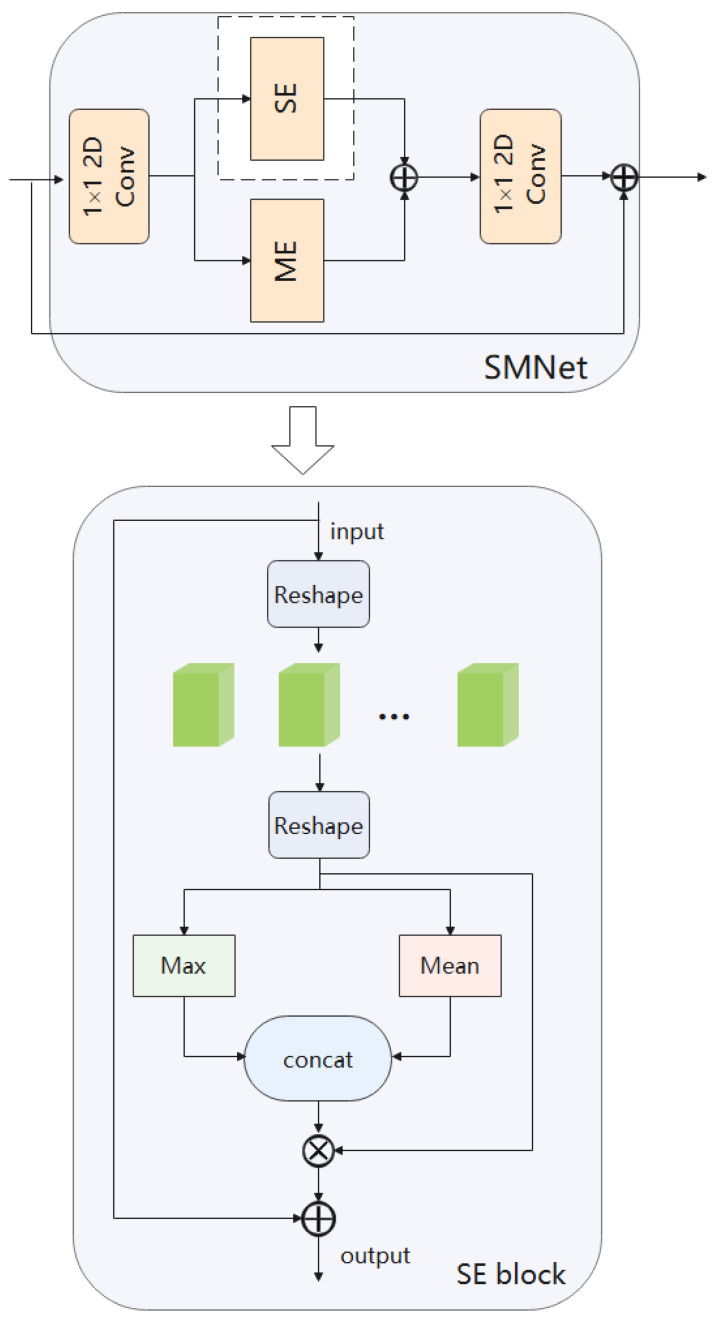
SE model. Using 1D group convolution acting in the time dimension to fuse information in the time domain. Using an attention mechanism on the spatial domain to learn about relationships on space.

Therefore, we incorporate group convolution and spatial attention mechanisms into our network framework. As shown in Figure 6, the use of 1D convolution on time dimension to fuse temporal information can be expressed as:

(1) $$F^{\prime }=Concate\left(F_{1},F_{2},\ldots ,F_{n}\right)$$

Here $F_{1},F_{2},\ldots ,F_{n}$ are the features obtained by convolution after being divided into *n* groups. Using the attention mechanism in the spatial domain to learn the spatial relationship, we use two pooling operations to aggregate the channel information of a feature graph. As shown in the Equation (2), each feature represents the average aggregation feature and maximum aggregation feature of the whole channel. The spatial attention matrix is extracted from the feature map of each layer on the spatial information path with rich location information, and the extracted spatial attention matrix is applied to the original feature map to determine the location that needs to be focused. Then we generate our two-dimensional spatial attention map through standard convolution layer connection and convolution. In short, spatial attention is calculated as follows:

(2) $$F_{1}^{*},\ldots ,F_{n}^{*}=Conv\left[AvgPool\left(F^{\prime }\right),MaxPool\left(F^{\prime }\right)\right]$$

(3) $$M_{s}\left(F_{*}\right)=Concate\left(F_{1}^{*},\ldots ,F_{n}^{*}\right)$$

Here, $F^{\prime }$ is the result of group convolution, and $F_{n}^{*}$ is the attention matrix obtained by each convolution. Our SMNet framework connects the two modules in series as shown in Equation (3), and finally performs a fusion. Our fusion operation can be expressed as Formula (4):

(4) $$G=F+F^{\prime }\times M_{s}\left(F_{*}\right)$$

#### 3.2.3. ME

**Figure 7:**
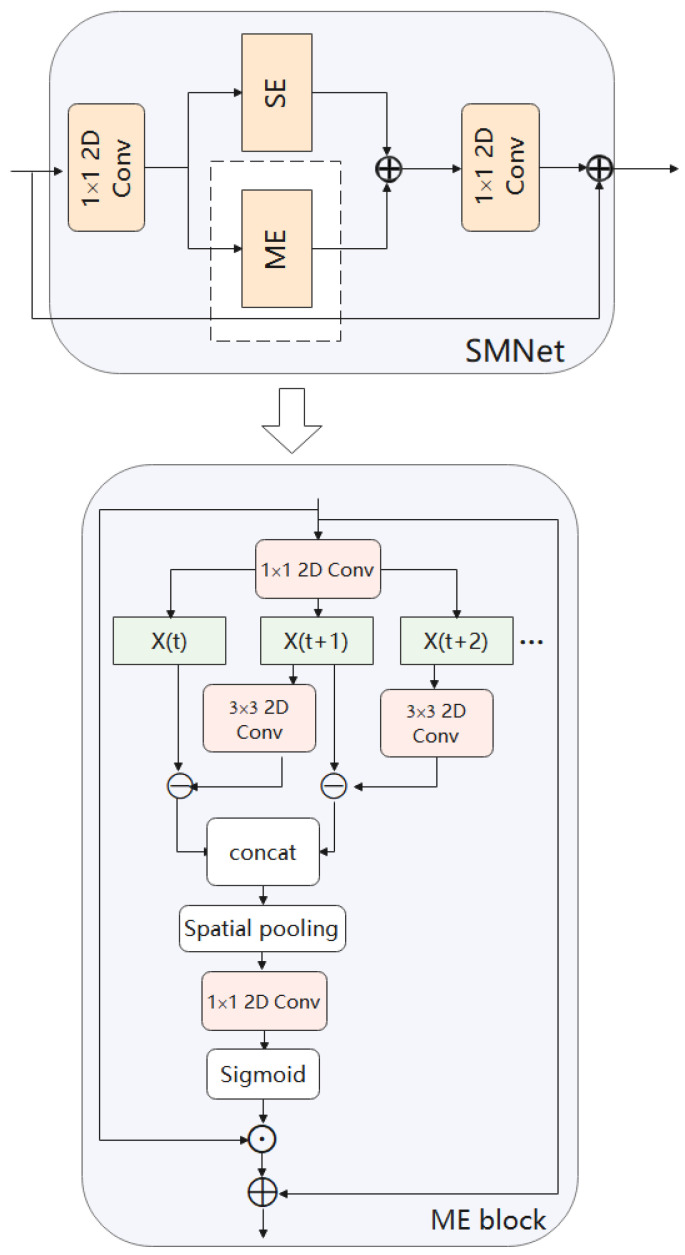
ME model. Use the difference between frames to represent motion information. In the temporal dimension, a difference operation is performed on two adjacent frames to produce T−1 motion representations. Then they are merged along the temporal channel to obtain the motion features.

ME module has been explored in [16,17]. It models motion information based on feature level. As a single RGB image usually encodes a static appearance and lacks contextual information about the previous frame and the next frame. An RGB difference operation between two consecutive frames, on the other hand, can describe changes in appearance, which may correspond to areas of motion prominence. In other words, we consider that among all feature channels, different channels capture different information. Some of the channels tend to model the static information related to the scene. Others are focused on describing dynamic information. For action recognition, the model should be made to focus more on these channels that are sensitive to dynamic information. The ME module is built based on this idea, and its structure is shown in Figure 7. Specifically, we consider that the feature-level motion representation at time step t can be approximated as the difference between two adjacent frames X(t) and X(t+1). We first perform a channel transformation on the features of X(t+1), and then use the transformed features to perform a difference. Formally this can be expressed as:

(5) M(t)=Conv3∗X(t+1)−X(t)1≤t≤T−1

where M(t) is the motion signature at time *t*. In this way, we can obtain a representation of our motion characteristics. In the temporal dimension, the difference operation can produce T−1 motion representations. Then they are merged along the temporal channel to obtain the motion features.

To obtain a classification task, we use cross-entropy as a loss function:

(6) $$L=\frac{1}{N}\sum_{i=1}^{T}\sum_{c=1}^{M}y_{ic}log\left(p_{ic}\right)$$

where *M* is the number of classes, *T* is the mini-batch size, $c=\left\{1,2,\ldots ,M\right\}$ is the true label of the ith sample, $p_{ic}$ is the predicted probability that observation sample *i* belongs to category *c*. $y_{ic}$ is a symbolic function, which is defined as:

$$y_{ic}=\left\{\begin{array}{cc} 0 & \mathrm{the}\;\mathrm{category}\;\mathrm{of}\;i\;\mathrm{is}\;c\\ 1 & \mathrm{the}\;\mathrm{category}\;\mathrm{of}\;i\;\mathrm{is}\;\mathrm{not}\;c \end{array}\right.$$

Different from other works [16,17], we use group convolution and spatial attention mechanisms in the SE module which we design to obtain more accurate spatio-temporal information, and we use the ME module in parallel with our proposed SE module. The results obtained from the two modules are fused at a later stage of the network, as shown in Figure 2. Finally, we discussed where to insert the SMNet module into ResNet-50 in order to make the most of our model. We can see the details of this operation in the experimental section.

## 4. Experiments and Results

### 4.1. Dataset

Something-Something V1 [29] contains 174 categories, including 86,017 training set videos, 11,522 verification set videos and 10,960 test set videos, with a total of 108,499 videos numbered from 1 to 108,499. Each video corresponds to a file, which contains jpg images with a height of 100 px and variable width jpg images are extracted from the original video at a rate of 12 frames per second. Jpg file names start with 00001.jpg. The number of jpgs vary with the length of the original video.

Something-Something V2 [29] is an extension of Something-Something V1. It contains 220,847 videos, including 168,913 in the training set, 24,777 in the verification set and 27,157 in the test set. A total of 174 tags show that humans perform predefined basic actions on daily objects.

Kinetics-400 [30] contains 400 categories, each category has at least 400 video clips, each clip is taken from different YouTube videos, lasting about 10 seconds, and provides 240 k training video and 20 k verification video. In our experiment, 223,127 training videos and 18,153 verification videos were used. For the Kinetics-400 dataset, the model is trained on the training set and evaluated on the validation set.

### 4.2. Implementation Details

#### 4.2.1. Training

According to the same strategy mentioned in TSN, we did experiments on the video action recognition task. For a given input video, we extracted T frames (T = 8 in our experiment) from each video segment according to the time average segmentation to obtain the input sequence, and the size of the shorter side of these frames is fixed to 256 and a central region of size 224 × 224 is cropped for action prediction. We pre-trained our model parameters on the ImageNet dataset [31]. Using two-dimensional ResNet-50 as the network backbone, our model is inserted into the residual block of ResNet for training. In addition, we use the data enhancement technology. During the training, we use random scaling and angle clipping to process the picture frame, and adjust the size of the input frame to 224 × 224.

#### 4.2.2. Testing

During the test, 10 different video clips were randomly sampled from the video, and the final prediction was obtained by the average of all clip scores. For each frame in the video clip, we follow the strategy proposed in [32], and adjust the smaller size to 256 by maintaining the aspect ratio. Then 3 crops of 256 × 256 that cover the full-frame are sampled for action prediction.

### 4.3. Experimental Results

Our model framework is compared with the existing action recognition methods on the datasets of Something-Something V1, Something-Something V2 and Kinetics-400. The results are shown in Table 1, Table 2 and Table 3. Here, top-1 (%) means that the classification with the maximum probability predicted is the probability of correct classification, and top-5 (%) is the probability that a correct classification exists among the five classifications with the highest predicted probability. We compared our results with the most advanced 2D CNN action recognition framework. Because the action recognition framework based on 3D CNN is slightly larger than 2D CNN and has more parameters, it is still a long way from industrial application, so we only compared a small portion of the 3D results.

Table 1 describes the accuracy of our model framework compared to existing 2D action recognition models and some 3D action recognition models on the Something-Something V1 dataset for the same condition of backbone and the same input frames. Our model is compared to a variety of 2D action recognition models with the input frames of 8, 16 and 8 + 16 respectively. When the input frames are 16, our model achieves a performance of 82.3% on top-5, 2% higher than TEA [16] and 1.9% higher than STM [17]. The performance of our model is also slightly higher when compared to partial 3D action recognition.

Table 2 describes some comparisons with the latest 2D action recognition models on the Something-Something V2 dataset. At the input frames of 8 + 16, our model achieves a performance of 91.9% on top-5, which is 2.1% higher than TEINet [15]. With the input frames of 8, our model achieves 63.2% performance on top-1, 1.9% higher than TEINet [15], but an accuracy of 87.6% on top-5, 1.2% lower than the best STM [17] model, which probably due to the smaller number of input frames.

Table 3 describes that our method compares with some of the more recent action recognition methods on the Kinetics-400 dataset. We can see that our accuracy is slightly lower compared to CorrNet [37] and S3D-G [36] on top-1 and top-5, this is due to the fact that both methods are 3D action recognition methods that handle the temporal information very well. In the 2D action recognition method, we can see that our model achieves a performance of 76.8% on top-1, which is almost the same as TANet [35]. This is probably due to the fact that our model does not handle the viewpoint problem well enough in the Kinetics-400 dataset, where the same action in different views is not well recognised and there is not a good focus on the rate aspect.

As the current state-of-the-art action recognition includes transformer-based systems, we have compared it with some advanced transformer models on the Something-Something V2 dataset. The results of the comparison are shown in Table 4.

We compared our experimental results on the Something-Something V2 dataset with those in some transformer models. We set our input sequence to 8 + 16 frames. The comparison results are shown in Table 4. We can find that we achieve a Top-5 of 91.9% on Something-Something V2, which is slightly higher than the transformer model. This may be due to the more complex temporal inference required in the Something-Something V2 dataset. However, since transformer is very computationally intensive, it is also not applicable in realistic scenarios.

**Figure 8:**
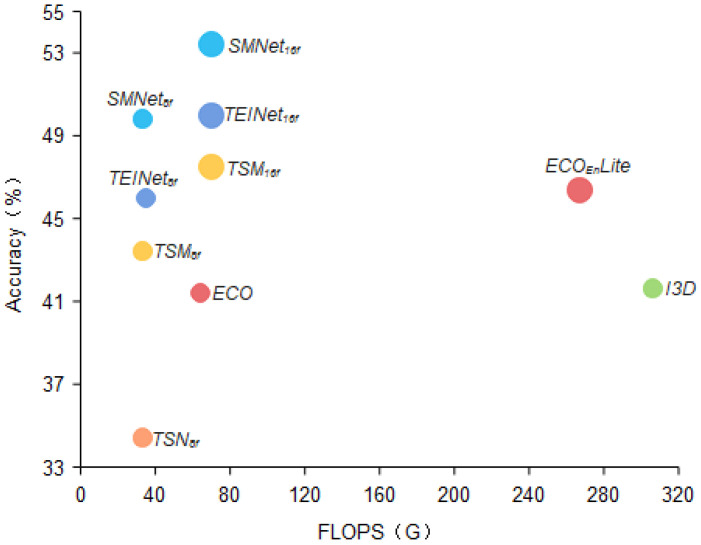
Video classification performance comparison. We compare in terms of Top-1 accuracy as well as computational cost for the Something-Something V1 dataset. We propose SMNet compares well with TEINet [11], TSM [12], ECO [34], and I3D [18] in terms of trade-offs between accuracy and efficiency.

We compare our SMNet model framework with several recent action recognition frameworks in terms of computational volume as well as number of parameters, and the results are shown in Table 5. We experiment with TSN, TSM, STM, I3D and our SMNet with backbone as resnet-50 and ECO [34], ECOEnLite [34] with backbone as BNInception+3D ResNet-18 on the Something-Something V1 dataset. We can clearly see from Figure 8 that our approach achieves a good trade-off in terms of number of parameters and flops, ensuring a small increase in parameters and computation while improving accuracy.

### 4.4. Ablation Study

In this section, we will conduct our ablation experiments on Something-Something V2 dataset and Something-Something V1 dataset. All experiments take the RGB images as input.

#### 4.4.1. Sampling Strategy

We discuss our a priori knowledge. To further demonstrate the effect of sparse sampling on our experiments, we conducted a comparison experiment between random and sparse sampling on the Something-Something V1 dataset. In random sampling, we cut the video and selected 8 random frames to obtain our input sequence. In sparse sampling, we split the video into 8 equal segments according to the chronological order of the action. One frame in each segment is randomly selected to obtain our input sequence. The experimental results are shown in Table 6. We can clearly see how sparse sampling helps the experiment.

Since the results of random sampling are not constant, the resulting sequences do not necessarily include the first, middle and second periods of the action. However, sparse sampling can model the video over a long range of time, and the resulting input sequence can contain all periods of action occurrence. Therefore, in our experiments we apply a sparse sampling strategy to the entire video frame.

#### 4.4.2. Insert Position

We discussed the impact of inserting the module into the location of ResNet-50 on the overall model performance on the Something-Something V2 dataset. We know that ResNet-50 can be divided into 6 phases i.e., $Conv1,res_{\_2},res_{\_3},res_{\_4},res_{\_5}$ and FC layer. We insert the SMNet module into ResNet-50’s $res_{\_2},res_{\_2,3},res_{\_2,3,4},res_{\_2,3,4,5}$ respectively to perform the insertion experiment. The experimental results are shown in Table 7. We set the baseline as the base ResNet-50 network. We can find that replacing the residual block with our module can significantly improve the accuracy of the experiment, and we replaced several residual blocks of ResNet-50 for experiments. Compared to baseline, inserting our module in the ResNet-50 network improves the accuracy on top-1 by 0.6%, 1%, 1.6% and 2.7% respectively. From the experimental results, it is obvious that replacing all residual blocks with our module has a greater impact on the accuracy of the experiment.

Our SMNet module is used to replace all residual blocks of the ResNet-50 backbone from $res_{\_2}$ to $res_{\_5}$. We conducted an ablation study to experiment the effect of inserting different numbers of SMNet modules at different locations on the experimental results. Specifically, we replaced all the ResNet blocks with different numbers of SMNet modules at a particular stage and kept all other stages unchanged. We conducted the experiments on the Something-Something V2 dataset with 8 input frames and the results are shown in Table 8.

**Figure 9:**
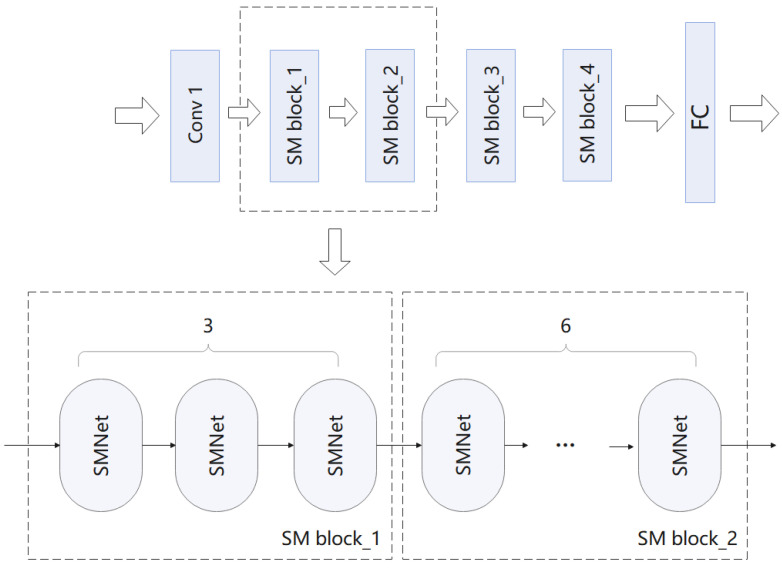
The way multiple SMNet modules are stacked in ResNet-50.

We can see from Table 8 that the insertion of SMNet modules into the later stages of action recognition is more helpful for recognition performance. The spatio-temporal features in the later stages will capture temporal information from a larger range and achieve the able temporal aggregation. Also, the performance of our method achieves the best results when the SMNet module is inserted into all phases of the ResNet backbone.We can see the way multiple SMNet modules are stacked in ResNet-50 in Figure 9.

#### 4.4.3. Fusion Strategy

We discussed the fusion pattern of our SE module and ME module on the Something-Something V2 dataset. In the experiment, we connected the SE module and ME module in parallel and series respectively. The experimental results are shown in Table 9. We can see that when two modules are connected in series, the accuracy is 61.7% on top-1 and 86.9% on top-5, but when two modules are connected in parallel, the accuracy is 63.2% on top-1 and 87.6% on top-5. We can clearly see that our accuracy will be better when our two sub modules are connected in parallel, so the subsequent experiments adopt the parallel strategy.

#### 4.4.4. Group Convolution

We discussed the influence of the number of groups of group convolution on the accuracy and computational effort of the model on the Something-Something V2 dataset. The results are shown in Table 10. We know that for group convolution, the more the number of groups, the better the experimental results. Therefore, we need to carry out some experiments to test the influence of the number of groups on the experiment. We can see that when the number of groups is 4, the performance trade-off of our model is better. When the number of groups exceeds 4, the increase of the number of groups will not be optimized in terms of accuracy and computation. This may be due to the limitation of hardware devices. If the hardware devices can keep up, we can try to increase the number of groups.

## 5. Conclusions

At present, the research on action recognition is still in full swing. Because the performance of 2D network is like that of 3D network, but it has less parameters and computation, 2D has better development prospects than 3D in industrial landing, and more and more researchers enter this field for research. Compared to existing 2D networks, this paper has a more accurate recognition performance and does not require pre-calculation of optical flow, reducing computational costs and working time. Compared to existing 3D networks, this paper is able to achieve the same performance as 3D networks without the need for excessive computational costs, which is of great interest in practical applications. In this paper we have also compared this with the state-of-the-art transformer model. However, the transformer is also not applicable in practical scenarios due to its very high computational effort. Overall, this paper presents an efficient spatio-temporal motion network that increases the model performance while minimizing the number of parameters and the computational effort of the model, which is of profound relevance for industrial use.

## Figures and Tables

**Table 1:** With input frames of 8, 16, 8 + 16, we compare with recent 2D action recognition frameworks and some 3D action recognition frameworks on the Something-Something V1 dataset.

Method	Backbone	Frames	Val Top-1 (%)	Val Top-5 (%)
2D CNNs:				
TSN-RGB [2]	ResNet-50	8	19.7	46.6
TSN-Multiscale [33]	BNInception	8	34.4	-
TSM-RGB [13]	ResNet-50	8	43.4	73.2
TEINet [11]	ResNet-50	8	47.4	-
STM-RGB [17]	ResNet-50	8	49.2	79.3
TEA [16]	ResNet-50	8	48.9	78.1
SMNet (ours)	ResNet-50	8	**49.8**	**79.6**
TSM-RGB [13]	ResNet-50	16	44.8	74.5
TEINet [11]	ResNet-50	16	49.9	-
STM-RGB [17]	ResNet-50	16	50.7	80.4
TEA [16]	ResNet-50	16	51.9	80.3
ECO [34]	BNInception+3D ResNet-18	16	41.4	-
SMNet (ours)	ResNet-50	16	**53.4**	**82.3**
TEINet [11]	ResNet-50	8 + 16	52.2	-
TSM [12]	ResNet-50	8 + 16	49.7	78.5
TANet [35]	ResNet-50	8 + 16	50.6	79.3
ECOEnLite [34]	BNInception+3D ResNet-18	92	46.4	-
SMNet (ours)	ResNet-50	8 + 16	**55.2**	**84.3**
3D CNNs:				
S3D-G [36]	Inception	64	48.2	78.7
I3D [18]	ResNet-50	64	41.6	72.2

**Table 2:** With input frames of 8, 16, 8 + 16, we compare with recent 2D action recognition frameworks on the Something-Something V2 dataset.

Method	Backbone	Frames	Val Top-1(%)	Val Top-5(%)
TSN-RGB [2]	ResNet-50	8	-	86.2
TSN-Multiscale [33]	BNInception	8	48.8	77.6
TSM [12]	ResNet-50	8	56.7	83.7
TEINet [11]	ResNet-50	8	61.3	-
STM [17]	ResNet-50	8	62.3	**88.8**
SMNet (ours)	ResNet-50	8	**63.2**	87.6
TSM [12]	ResNet-50	16	58.7	84.8
TEINet [11]	ResNet-50	16	62.1	-
STM [17]	ResNet-50	16	64.2	89.8
TEA [16]	ResNet-50	16	64.5	89.8
SMNet (ours)	ResNet-50	16	**65.7**	**90.1**
TEINet [11]	ResNet-50	8 + 16	65.5	89.8
TSM Two-Stream [12]	ResNet-50	8 + 16	63.5	88.6
SmallBigNet [38]	ResNet-50	8 + 16	63.3	88.8
SMNet (ours)	ResNet-50	8 + 16	**67.8**	**91.9**

**Table 3:** With different numbers of input frames, we compare with recent 2D action recognition frameworks and some 3D action recognition frameworks on the Kinetics-400 dataset.

Method	Backbone	Frames	Val Top-1(%)	Val Top-5(%)
2D CNNs:				
SmallBigNet [38]	ResNet-50	8 × 3 × 10	76.3	92.5
TEINet [11]	ResNet-50	16 × 3 × 10	76.2	92.5
TEA [16]	ResNet-50	16 × 3 × 10	76.1	92.5
TSM [12]	ResNet-50	16 × 3 × 10	74.7	91.4
TANet [35]	ResNet-50	16 × 4 × 3	**76.9**	92.9
TSN [2]	InceptionV3	25 × 10 × 1	72.5	90.2
R (2+1) D [39]	ResNet-34	32 × 1 × 10	74.3	91.4
TAM [40]	bLResNet-50	48 × 3 × 3	73.5	91.2
SMNet (ours)	ResNet-50	8 × 3 × 10	76.1	92.7
SMNet (ours)	ResNet-50	16 × 3 × 10	76.8	**93.3**
3D CNNs:				
SlowOnly [22]	ResNet-50	8 × 3 × 10	74.8	91.6
ARTNet [41]	ResNet-18	16 × 10 × 25	70.7	89.3
NL I3D [32]	ResNet-50	128 × 3 × 10	76.5	92.6
CorrNet [37]	ResNet-50	32 × 1 × 10	**77.2**	-
S3D-G [36]	InceptionV1	64 × 3 × 10	74.7	**93.4**

**Table 4:** With input frames 8 + 16. We have compared this with the state-of-the-art transformer model on the Something-Something V2 dataset.

Method	Pretrain	Val Top-1(%)	Val Top-5(%)
VidTr-L [42]	IN-21K+K-400	60.2	-
Tformer-L [43]	IN-21K	62.5	-
ViViT-L [44]	RIN-21K+K-400	65.4	89.8
MViT-B [45]	K-400	67.1	90.8
Mformer [46]	IN-21K+K-400	66.5	90.1
Mformer-L [46]	IN-21K+K-400	**68.1**	91.2
Mformer-HR [46]	IN-21K+K-400	67.1	90.6
SMNet (ours)	ImgNet	67.8	**91.9**

**Table 5:** The calculation amount of the model and the comparison of parameters on Something-Something V1 dataset.

Method	Backbone	Frames	FLOPs	Param
TSN [2]	ResNet-50	8	33 G	24.3 M
TSM [12]	ResNet-50	8	32.9 G	23.9 M
STM [17]	ResNet-50	8	33.3 G	24.0 M
ECO [34]	BNInception+3D ResNet-18	16	64 G	47.5 M
ECOEnLite [34]	BNInception+3D ResNet-18	92	267 G	150 M
I3D [6]	ResNet-50	64	306 G	28.0 M
SMNet (ours)	ResNet-50	8	33.1 G	23.9 M

**Table 6:** Comparative results of random and sparse sampling. We conduct experiments on the Something-Something V1 dataset. For random sampling, we sampled 8 frames in the whole segment. For sparse sampling, we divide the video equally into 8 segments and sample each segment randomly.

Sampling Strategy	Number of Segments	Number of Input Sequence Frames	Val Top-1 (%)
Random sampling	1	8	45.7
Sparse sampling	8	8	49.8

**Table 7:** Test the module in different positions of ResNet on the Something-Something V2 dataset. The SMNet module is inserted into the $res_{\_2}$ residual block, the $res_{\_2}$ and $res_{\_3}$ residual blocks, the $res_{\_2}$, $res_{\_3}$ and $res_{\_4}$ residual blocks and the $res_{\_2}$, $res_{\_3}$, $res_{\_4}$ and $res_{\_5}$ residual blocks of ResNet to compare the impact of the insertion position and the number of modules inserted on the accuracy.

Stage	Top-1 (%)	Top-5 (%)
baseline	60.5	85.3
$res_{\_2}$	61.1	85.7
$res_{\_2,3}$	61.5	86.3
$res_{\_2,3,4}$	62.1	86.8
$res_{\_2,3,4,5}$	63.2	87.6

**Table 8:** We inserted different numbers of SMNet blocks at different locations. At one stage we replaced all the ResNet blocks with a different number of SMNet modules, the other stages remained the same.

Stage	Number of the SMNets	Val Top-1(%)
$res_{\_2}$	3	61.1
$res_{\_3}$	4	61.3
$res_{\_4}$	6	61.9
$res_{\_5}$	3	61.5
$res_{\_2,3,4,5}$	16	63.2

**Table 9:** Compare the fusion strategies between modules on the Something-Something V2 dataset. Operate the SE and ME modules in parallel and in series respectively to compare accuracy.

Fusion Strategy	Top-1 (%)	Top-5 (%)
serial	61.7	86.9
parallel	63.2	87.6

**Table 10:** Experiment on the number of groups of group convolution on the Something-Something V2 dataset.

Group Number	FLOPs	Top-1 (%)	Top-5 (%)
2	33.4 G	62.8	87.1
4	33.1 G	63.2	87.6
6	33.1 G	63.1	87.4
8	33.1 G	62.9	87.5

## Data Availability

Not applicable.
